# Small molecule MMRi62 targets MDM4 for degradation and induces leukemic cell apoptosis regardless of p53 status

**DOI:** 10.3389/fonc.2022.933446

**Published:** 2022-08-05

**Authors:** Rati Lama, Chao Xu, Samuel L. Galster, Javier Querol-García, Scott Portwood, Cory K. Mavis, Federico M. Ruiz, Diana Martin, Jin Wu, Marianna C. Giorgi, Jill Bargonetti, Eunice S. Wang, Francisco J. Hernandez-Ilizaliturri, Gerald B. Koudelka, Sherry R. Chemler, Inés G. Muñoz, Xinjiang Wang

**Affiliations:** ^1^ Department of Pharmacology and Therapeutics, Roswell Park Comprehensive Cancer Center, Buffalo, NY, United States; ^2^ Department of Chemistry, University at Buffalo, The State University of New York, Buffalo, NY, United States; ^3^ Structural Biology Programme, Spanish National Cancer Research Centre (CNIO), Madrid, Spain; ^4^ Department of Medicine, Roswell Park Comprehensive Cancer Center, Buffalo, NY, United States; ^5^ The Department of Biological Sciences, Hunter College, City University of New York, New York, NY, United States; ^6^ Department of Biological Sciences, University at Buffalo, The State University of New York, Buffalo, NY, United States

**Keywords:** MDM2, MDM4, E3 ligase, degradation, apoptosis, leukemia, ubiquitination, p53

## Abstract

MDM2 and MDM4 proteins are key negative regulators of tumor suppressor p53. MDM2 and MDM4 interact *via* their RING domains and form a heterodimer polyubiquitin E3 ligase essential for p53 degradation. MDM4 also forms heterodimer E3 ligases with MDM2 isoforms that lack p53-binding domains, which regulate p53 and MDM4 stability. We are working to identify small-molecule inhibitors targeting the RING domain of MDM2-MDM4 (MMRi) that can inactivate the total oncogenic activity of MDM2-MDM4 heterodimers. Here, we describe the identification and characterization of MMRi62 as an MDM4-degrader and apoptosis inducer in leukemia cells. Biochemically, in our experiments, MMRi62 bound to preformed RING domain heterodimers altered the substrate preference toward MDM4 ubiquitination and promoted MDM2-dependent MDM4 degradation in cells. This MDM4-degrader activity of MMRi62 was found to be associated with potent apoptosis induction in leukemia cells. Interestingly, MMRi62 effectively induced apoptosis in p53 mutant, multidrug-resistant leukemia cells and patient samples in addition to p53 wild-type cells. In contrast, MMRi67 as a RING heterodimer disruptor and an enzymatic inhibitor of the MDM2-MDM4 E3 complex lacked MDM4-degrader activity and failed to induce apoptosis in these cells. In summary, this study identifies MMRi62 as a novel MDM2-MDM4-targeting agent and suggests that small molecules capable of promoting MDM4 degradation may be a viable new approach to killing leukemia cells bearing non-functional p53 by apoptosis.

## Introduction

Leukemia, which was an incurable disease a century ago, is now highly manageable with cure rates ranging from 30% to >90% for the different subtypes (1, 2). This remarkable achievement can be attributed to the development of new chemotherapeutics, drug combinations, adjusted dose/scheduling regimens, and various targeted therapies. In particular, leukemia outcomes have been transformed by targeted therapies based on all-trans retinoic acid for the retinoic acid receptor alpha (RARα), arsenic trioxide for promyelocytic leukemia (PML)-RARα, imatinib for the *BCR-ABL* fusion gene, and ibrutinib for Bruton’s tyrosine kinase (RTK), as well as antibody-based therapies such as rituximab for CD-20 and gemtuzumab ozogamicin for CD33 (1, 2). In difficult-to-treat leukemia subtypes, new targeted therapies against FMS-like tyrosine kinase 3 (FLT3) and isocitrate dehydrogenase 1 and 2 (IDH1/IDH2), as well as venetoclax for B-cell lymphoma 2 (BCL-2), also have brought about improved long-term survival in recent years (2–5). Despite this tremendous progress, leukemia remains a major type of malignancy that contributes to ~24,000 estimated cancer deaths in the United States (6). Further progress will necessitate new targeted therapies that can improve outcomes for difficult-to-treat leukemias such as acute myeloid leukemia (AML) and other subtypes with frequent recurrence that no longer respond to current therapies.

Apoptosis is the predominant mechanism of DNA-damaging chemotherapies that kill leukemia cells (7), and evasion of apoptosis is known to contribute to the progression of cancer and resistance to therapy (8). For example, overexpression of BCL-2, an X-linked inhibitor of apoptosis protein (XIAP), and myeloid cell leukemia 1 (MCL-1) blocks the intrinsic apoptotic pathway and confers resistance to chemotherapy in leukemia (9–12). In addition to the involvement of BCL-2, XIAP, and MCL-1 (13), p53 mutant leukemia remains a challenge to treat because p53 positively regulates apoptosis by upregulating PUMA and NOXA, the pro-apoptotic BH3 (BCL-2 homology 3)-only members of the BCL-2 family (14), and these pathways may also play a role in disease progression. A TP53 mutation occurs in ~13% of AML cases (15), and this mutation is an independent prognosis factor for a lower response rate, inferior complete remission duration, and overall survival in chronic lymphocytic leukemia (CLL) (16), AML (17), myelodysplastic syndromes (18), and an ultra-high-risk group of relapsed pediatric T-cell acute lymphoblastic leukemia (TALL) patients (19).

MDM2 and MDM4 are established cancer drug targets since they are inhibitors of p53 activity and are amplified in many cancer types (6, 20–22). In particular, the targeting of MDM2–p53 interactions is a strategy that is being actively pursued to unleash the antitumor activity of p53. Currently, multiple, potent, small-molecule inhibitors of MDM2–p53 interactions are in clinical trials with hematological malignancies as the primary targets (23). However, these inhibitors are not designed to work on p53 mutant leukemia. We previously reported that MDM2 does not act alone in regulating p53, and this regulatory process requires interaction with the MDM4 RING (really interesting new gene) domain to form a polyubiquitin E3 ligase that promotes p53 degradation (24). Consistent with this biochemical mechanism, genetic studies have found that the RING domains of both MDM2 and MDM4 are required for the two oncoproteins to restrict p53 activity *in vivo* (25–27). We also reported that MDM4 forms heterodimer E3 ligases with splice isoforms MDM2A and MDM2B, which lack p53 binding domains (28). In a recent genetic study using a *Mdm2^L466A^* mouse model that genetically separates E3 ligase-dependent functions from E3 ligase-independent functions of MDM2-MDM4 heterodimer, we further demonstrated that the E3 ligase activity of MDM2-MDM4 is essential not only for p53 regulation but also for timely G2/M cell cycle transition independent of p53 (29). Therefore, targeting the RING domains of MDM2-MDM4 may deliver broader antitumor effects since this will potentially inactivate all the E3 ligase activities of MDM4-MDM2, MDM4-MDM2A, and MDM4-MDM2B, thereby unleashing their downstream substrates including p53. In earlier work, we performed a high-throughput screen for the identification of MDM2-MDM4 RING domain inhibitors (designated as MMRi) and demonstrated that MMRi64 has higher p53-dependent pro-apoptotic activities than MDM2–p53 inhibitors (30). In this study, we report on the identification and characterization of small-molecule MMRi62 as an MDM4 degrader and a p53-independent apoptosis inducer with the potential to overcome daunorubicin resistance in p53 null leukemia cells.

## Materials and methods

### Cell culture and chemical compounds

All the leukemic cell lines were cultured in RPMI-1640 medium supplemented with 10% fetal bovine serum and 50 U/ml of penicillin and 50 μg/ml of streptomycin. NALM6 (wt-p53, acute lymphoblastic leukemia (ALL)) and HL60 (AML, p53-null) were from the American Type Culture Collection (Manassas, VA, USA). MV4-11 (wt-p53, AML), Jurkat (p53-null, T-cell leukemia), and CCRF-CEM (p53-mut ALL) were gifts from Dr. John McGuire. NALM6shp53 cell line was established with recombinant lentivirus expressing pLKO.1-shp53 DNA (#19119 (31); Addgene, Watertown, MA, USA) followed by puromycin selection at 1 μg/ml for 2 days and then expansion in fully supplemented RPMI-1640 medium. Transfection was carried out with Lipofectamine™ 2000 (Invitrogen, Washington, DC, USA). MANCA, MANCA-mlp-puro, and MANCA-mlp-MDM2 were generated as described previously (32) and maintained in 10%FBS-Pen/Strep-RPMI-1640 medium. Small-molecule compounds MMRi62 and MMRi67 were synthesized in-house. The Betti reaction was used for MMRi62 and MMRi67 syntheses as described in the literature (33, 34), and the details and their characterization are provided in the **Supplementary Methods**. Other MMRi derivatives in the secondary screening were purchased from Hit2Lead ChemBridge Chemical Store (San Diego, CA, USA). The compounds were dissolved in dimethyl sulfoxide (DMSO) as 10 mM stocks. SPYRO Orange dye was purchased from Thermo Fisher (Waltham, MA, USA) and used in ThermoFluro and microscale thermophoresis (MST) assays.

### Plasmids, antibodies, and primers

HA-FLAG-MDM4, HA-MDM2, and HA-MDM2B plasmids for insect cell and mammalian expression were described previously (24, 28). His-ubiquitin plasmid (pMT107) was a gift from Dr. Dirk P. Bohmann (University of Rochester Medical Center, Rochester, NY, USA). MDM2-MDM4 RING heterodimer constructs, pETDuet-MDM2R, and pETDuet-MDM4R were generated by PCR cloning of the RING domain of human MDM2 or MDM4 into pETDuet-1 (Novagen, Madison, WI, USA). Plasmid shp53 pLKO.1 puro was from Addgene (31). Detailed information on antibodies for Western blotting (WB) analysis as well as primer sequences and conditions for RT-PCR analysis of gene expression can be found in the **Supplementary Methods**.

### *In vitro* and *in vivo* ubiquitination

*In vitro* assays for ubiquitination by MDM2-MDM4 were performed as described previously (24). Briefly, reactions were carried out at 30°C for 1 h in a reaction volume of 20 μl in the presence of different concentrations of MMRi or vehicle solvent DMSO, followed by WB of p53 with DO-1, MDM2 with anti-HA, MDM4 with a rabbit anti-MDM4 antibody, and polyubiquitin with an anti-ubiquitin. *In vivo* ubiquitination was performed as described previously (28). Briefly, 293 cells were transfected with pcDNA3.1-MDM2B and FLAG-MDM4, pEGFP with or without His-ubiquitin plasmid. Sixteen hours after transfection, cells were treated with 5 and 10 μM of MMRi62 or MMRi67 for 24 h before denatured His-pulldown of the proteins followed by WB for MDM2 and MDM4.

### Biochemical and biophysical analyses of the compound effect on RING–RING domain interactions

*In vitro* pulldown assays using insect cell-expressed and affinity-purified FLAG-MDM4 and HA-MDM2B were performed as described previously (30). Briefly, after incubation of FLAG-MDM4 and HA-MDM2B proteins in the presence of different concentrations of compounds, FLAG-MDM4 was pulled down by anti-FLAG beads followed by multiple washing steps with wash buffer. MDM4-bound MDM2 was detected by WB with an anti-HA antibody. RING heterodimers of MDM2-MDM4 were used in MST and Thermofluor assays. RING heterodimers of MDM2-MDM4 were expressed and purified in *Escherichia coli* cells by a two-step method with chromatography using a HisTrap column and Superdex 75 columns as detailed in the **Supplementary Methods**. MST as detailed in the **Supplementary Methods** was used to determine the binding affinity of MMRi62 and MMRi67 on preformed RING heterodimers. Briefly, the RING complex was labeled with DY-547P1 dye, and compounds MMRi62 and MMRi67 were added to wells in 16 serial dilutions for final concentrations ranging from 100 µM to 3 nM; all samples were run together at a Monolith NT.115 in duplicates for every set of measurements. Kd ± standard errors and the fitting graphs were derived by using MO.Affinity Analysis v2.2. Thermofluor assays were performed as detailed in the **Supplementary Methods** to assess the effects of compounds on the thermal stability of MDM2-MDM4 RING heterodimers under conditions containing RING heterodimers at a final concentration of 33 µM mixed with MMRi62 or MMRi67 compounds at final concentrations of 5, 10, and 20 µM in buffer consisting of 500 mM of NaCl, 50 mM of Tris (pH 8), and 1 mM of Tris(2-carboxyethyl)phosphine hydrochloride (TCEP).

### Docking analysis

The Sybyl X 2.2 (Tripos Inc.) software package and crystal structure of the MDM2-MDM4 RING domain heterodimer (PDB code: 2VJF) were used for the docking analysis, as detailed in the **Supplementary Methods**. The ligands were docked using the flexible protein feature, in which protein heavy atom and hydrogen shifts were enabled. Amino acid residues of interest and potential hydrogen bonds were displayed.

### IC50 measurement

Cells at 5,000–10,000/well were plated in 96-well plates at 100 µl/well, and compounds of different concentrations at double dilutions with the corresponding medium were added to each well at 100 µl/well. After the cells were cultured for 70 h, 40 µl of 6× resazurin stock solution was added to each well to allow for the formation of fluorescent metabolites by viable cells for 2 h, followed by readings at an optical density of 600 nm (OD600) in a BioTek Synergy 2 Microplate Reader. The IC50 (half maximal inhibitory concentration) values were obtained by the Chou-Median-Effect Equation using CompuSyn software (35), and dose–effect curves were obtained by GraphPad using the affected fractions of compound-treated wells normalized against no-drug control wells with a non-linear regression model.

### Apoptosis analysis by flow cytometry

NALM6 cells were treated with MMRi62 or MMRi67 for 48 h before flow cytometry analysis. Primary AML patient cells were grown in Serum-Free IMDM supplemented with essential factors as detailed in the **Supplementary Methods**. After cells were treated in triplicate at a concentration of 1 × 10^5^ cells/ml in 24-well plates for 72 h, cells were processed with DNA staining dye 7-amino-actinomycin (7-AAD) and FITC-annexin-V as described by the manufacturer’s instructions, and apoptotic cells were measured by flow cytometry.

### Colony-forming unit assays with acute myeloid leukemia patient samples

AML patient samples were obtained from multiple patients under institutional review board (IRB)-approved protocols from the Roswell Park Hematologic Procurement Shared Resource. Leukemic cells were treated for 4 h with compounds at different concentrations in a 37°C–5% CO_2_ incubator at 360,000 cells/450 µl of WMB (120,000 cells/150 µl) per condition. The ingredients of WMB are detailed in the **Supplementary Method**. After 4 h of drug treatment, 3.1 ml of MethoCult, a semisolid methylcellulose medium for the optimal growth of hematopoietic progenitor cells, was added to the 450 µl cell suspension, and then, the MethoCult suspension was spread out among three wells at 1 ml/well in a 6-well plate for 12–15 days. Colonies were quantified using a Spot-RT3 camera on an inverted microscope with SPOT-Basic imaging software. The MMRi62 IC50s were derived from the average colonies of three wells for each drug concentration for each patient sample treated with 1, 10, 25, and 50 µM.

## Results

### Identification of MMRi62 and MMRi67 with distinct mechanisms in the inhibition of MDM2-MDM4 E3 ligase and apoptosis induction

**Figure 1:**
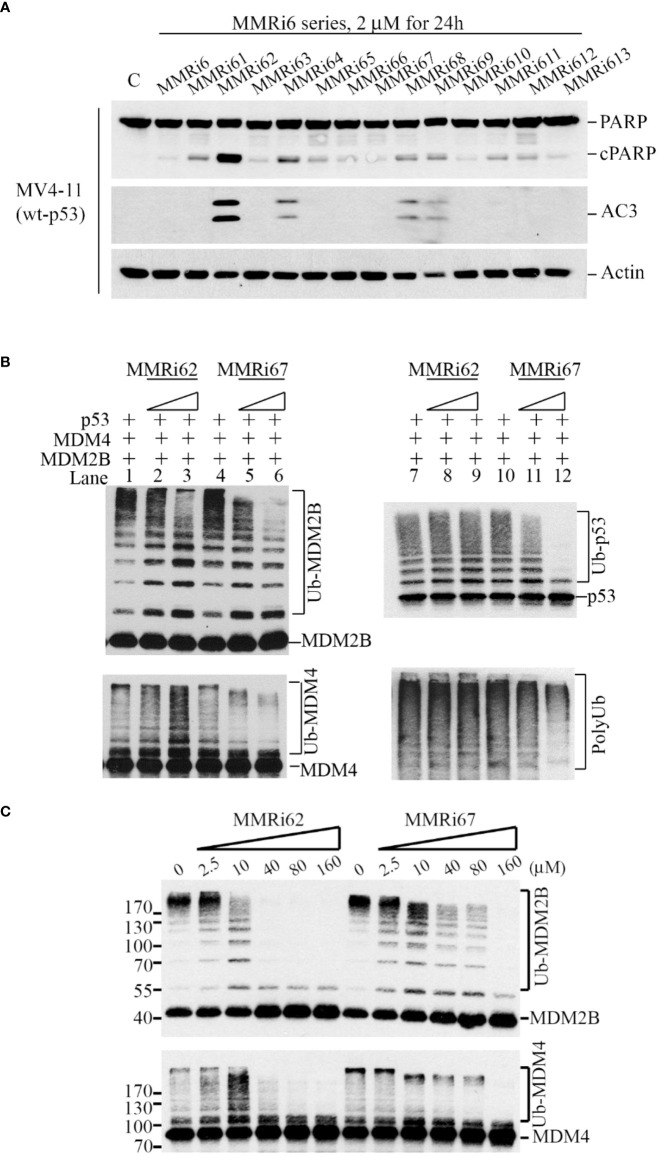
Identification of MMRi62 as an apoptosis inducer and MMRi67 as E3 ligase inhibitor by apoptosis and E3 ligase inhibitor screens. **(A)** Apoptosis screen of MMRi6 analogs by activated caspase 3 (AC3) and cleaved PARP as readouts using wt-p53 bearing MV4-11 cells treated with 2 μM of MMRi6 analogs for 24 h. **(B)** *In vitro* ubiquitination assay using recombinant MDM2B, MDM4, and p53 proteins in the presence of the solvent dimethyl sulfoxide (DMSO) or MMRi6 analogs (0, 5, and 10 μM of MMRi62 or MMRi67); ubiquitinated species of MDM2B, MDM4, p53, and polyubiquitin are shown. **(C)** *In vitro* ubiquitination assays as performed in panel B using MDM4 and MDM2B with an extended range of concentrations of MMRi62 and MMRi67; ubiquitinated species of MDM2B and MDM4 are shown.

We previously reported on the identification of several MMRi hits in high-throughput screening and demonstrated that MMRi64 activates the p53 pathway with preferential induction of apoptosis in leukemia/lymphoma cells (30). To identify better apoptosis inducers among MMRi6 analogs, we screened all available MMRi6 derivatives with a cell-based apoptotic assay using activated caspase 3 and poly (ADP-ribose) polymerase (PARP) cleavage as readouts. MMRi62 was identified as the best analog for inducing apoptosis in a broad range of leukemia/lymphoma cell lines (**Figure 1A** and data not shown). However, the E3 ligase activity-based screen using *in vitro* p53 ubiquitination by MDM2-MDM4 heterodimers identified MMRi67 as the most potent E3 ligase inhibitor of the MDM2-MDM4 E3 complex and MMRi62 as a weak inhibitor (**Figure S1A**). Thus, we further characterized the effects of these two compounds by using splice isoform MDM2B that lacks p53-binding domains but can lead to the formation of super E3 ligase activity toward p53, MDM4, and MDM2, as described in our previous studies (28). When MMRi62 and MMRi67 at two concentrations (5 and 10 µM) were compared in the presence or absence of p53, we found that MMRi62 decreased MDM2B autoubiquitination (**Figure 1B**, left top, lanes 1–3) and increased MDM4 ubiquitination (**Figure 1B**, left bottom, lanes 1–3) without a significant effect on p53 ubiquitination (**Figure 1C**, right top, lanes 7–9) or total polyubiquitination (**Figure 1B**, right bottom, lanes 7–9). In contrast, MMRi67 inhibited ubiquitination of every component of the ternary complex, MDM2, MDM4, and p53, in a dose-dependent manner (**Figure 1B**, left lanes 4–6 and right lanes 10–12). Similar effects of MMRi62 and MMRi67 were obtained in E3 ligase assays in the absence of p53 protein (**Figure S1B**). These results suggest that MMRi62 is an E3 ligase modifier capable of switching substrate preference from MDM2 to MDM4, while MMRi67 acts as an E3 ligase inhibitor of MDM2-MDM4. To better understand the differences in their mechanism of action, we performed E3 ligase assays with a broader range of compound concentrations up to 160 µM. Our results showed that the MMRi62 treatment produced two distinct patterns of ubiquitination of MDM2B and MDM4 in a concentration-dependent manner (**Figure 1C**), i.e., preferential MDM4 ubiquitination coincided with reduced MDM2 autoubiquitination at concentrations below 10 µM, and decreased ubiquitination of both MDM2 and MDM4 was observed at concentrations above 10 µM (**Figure 1C**, left). These results suggest that MMRi62 has RING domain modifier activity as a dominating mechanism of action at concentrations below 10 µM and non-selective E3 ligase inactivating activity at concentrations above 10 µM. Notably, MMRi62 at concentrations of 40 µM and above caused abrupt loss of E3 ligase activity, similar to MMRi67 at ≥40 µM. In contrast, MMRi67 generated a single ubiquitination pattern of decreased ubiquitination of both MDM2 and MDM4 in a concentration-dependent manner (**Figure 1C**). These results suggest that MMRi62 and MMRi67 have distinct mechanisms of action on the E3 ligase activity of MDM2-MDM4 E3 ligases.

**Figure 2:**
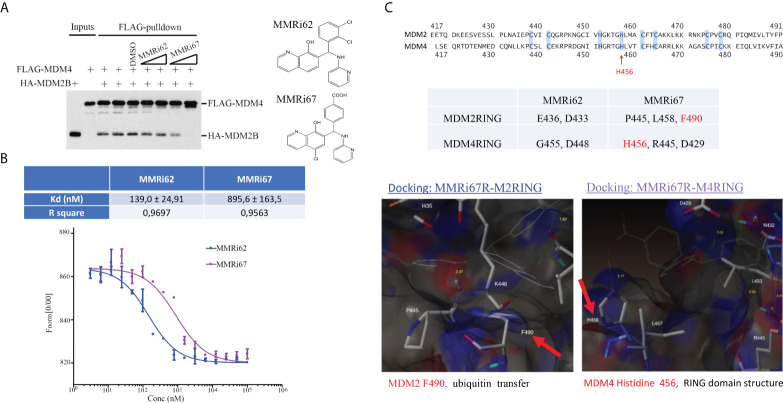
Characterization of drug–target interactions *in vitro*. **(A)** Left, *in vitro* pulldown assay with recombinant FLAG-MDM4 and HA-MDM2B in the presence of solvent dimethyl sulfoxide (DMSO) or 5 and 10 μM of MMRi62 or MMRi67. After anti-FLAG pulldown, the FLAG-MDM4-bound HA-MDM2B was detected by Western blotting (WB) with anti-HA antibody. Right, chemical structures of MMRi62 and MMRi67. **(B)** Measurement of the binding affinity of MMRi62 and MMRi67 to preformed RING–RING heterodimers of MDM2 and MDM4 in microscale thermophoresis (MST) analyses. MST analyses were performed in the presence of MMRi62 or MMRi67 at concentrations ranging from 3 nM to 100 μM obtained through serial dilutions. The fluorescence intensities (y-axis, Fnorm%) were normalized to the overall highest detected signal. The equilibrium dissociation constant between the MMRi62/MMRi67 and RING heterodimers (Kd) is presented. Top, the calculated Kd for MMRi62 and MMRi67; bottom, the fitting curve of the measurements using MO.Affinity Analysis software. **(C)** Molecular modeling of interaction interfaces for the RING domains of MDM2 and MDM4 with MMRi62 and MMRi67. Top, amino acid alignment of RING domains of MDM2 and MDM4. Middle, the interaction residues of RING domains with MMRi62 and MMRi67 identified by docking analysis. The F490 region in the MDM2 RING is critical for E3 ligase activity of MDM2, and the H456 region in the MDM4 RING is critical for chelating a Zn to maintain the MDM4 RING domain structure. Bottom, presentations of the docking interface—the results for the MMRi67R enantiomer are shown.

To understand how MMRi62 and MMRi67 affect RING domain heterodimer interactions of MDM2 and MDM4, we performed *in vitro* pulldown experiments by incubating the compounds at two concentrations with FLAG-MDM4 and HA-MDM2B recombinant proteins. MMRi62 did not inhibit RING–RING interactions of MDM4 and MDM2B in solution, but MMRi67 inhibited these interactions in a dose-dependent manner (**Figure 2A**). Then, we measured the binding affinity of the two compounds to preformed RING domain heterodimers by MST analysis and obtained a Kd of ~140 nM for MMRi62 and ~896 nM for MMRi67 (**Figure 2B**). These data suggest that MMRi62 is a better binder but has a weaker effect on RING heterodimer formation, while MMRi67 is a weaker binder to heterodimer RINGs but has a stronger effect on the inhibition of *de novo* RING heterodimer formation. To obtain insights into the interaction interface of the two compounds with RING domains of MDM2 and MDM4, we performed molecular modeling by a docking analysis of the two compounds against the crystal structure of RING domain heterodimers. Individual RING domain structures were derived from the RING domain heterodimers and used for docking under flexible ligand and rigid protein conditions. We used R-enantiomers of the compounds for docking because these retain the activity of a racemic mixture (data not shown). Our analysis showed that MMRi62 R-enantiomer forms hydrogen bonds with E436 and N433 of the MDM2RING and G455 and N448 of the MDM4 RING (data not shown). Of note, none of these hydrogen bonding residues were involved in E2 binding or heterodimer formation, which is consistent with the expected interface. In contrast, results showed that MMRi67 R-enantiomer forms hydrogen bonds with P445 and L458 and a p-stack of F490 of the MDM2RING (**Figure 2C**, bottom left) and H456, R445, and D429 of the MDM4 RING (**Figure 2C**, bottom right). Since H456 is a critical residue for Zn-chelation, which maintains the RING domain structure, MMRi67 interaction with H456 of the MDM4 RING domain is predicted to disrupt the RING domain, thus abolishing its ability to interact with the RING domain of MDM2, which is consistent with the MMRi67 effect in the RING domain pulldown assays (**Figure 2A**).

### Induction of MDM2-dependent MDM4 degradation in cells by MMRi62

**Figure 3:**
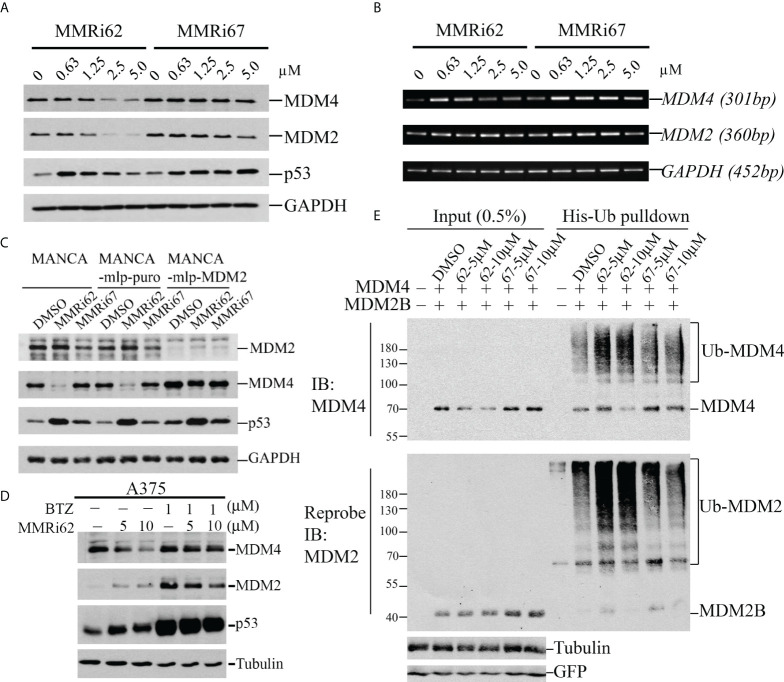
MMRi62 induces MDM2-dependent degradation of MDM4 protein in cells. **(A)** Western blotting analysis of the effects of MMRi62 and MMRi67 on expression levels of p53, MDM2, and MDM4 in NALM6 cells treated for 24 h at the indicated concentrations. **(B)** RT-PCR analysis of the effects of MMRi62 and MMRi67 on *p53*, *Mdm2*, and *Mdm4* mRNA expression in the same NALM6 samples prepared in panel **(A)**. **(C)** Results demonstrating that MDM2 is required for MMRi62-induced degradation of MDM4 proteins in cells. The Western blotting (WB) analysis was performed as in panel A except in MANCA cells, or cells stably expressing empty vector mlp (MANCA-mlp-puro) or stably expressing MDM2-mcRNA (MANCA-mlp-MDM2); cells were treated with 5 μM of MMRi62 or MMRi67. **(D)** Western blotting analysis of MDM4 degradation by MMRi62 rescued by proteasome inhibition with bortezomib (BTZ); in these experiments, A375 cells were treated with MMRi62 for 24 h with or without BTZ for 16 h before cell harvest. **(E)** MMRi62 induced increased ubiquitination of MDM4 and MDM2 in 293 cells during *in vivo* ubiquitination assays. The 293 cells were transfected with vectors expressing MDM2B, FLAG-MDM4, His-ubiquitin, and green fluorescent protein (GFP). Cell lysates were prepared 24 h after transfection and used for His-tag pulldown followed by WB with anti-FLAG (for MDM4) and anti-MDM2 (mAb 4B11). Ubiquitinated MDM4 and MDM2B are indicated. GFP was used for the internal control of transfection efficiency and protein inputs.

We performed a WB analysis of NALM6 cells treated with MMRi62 and MMRi67 at different concentrations for 24 h to test their effect on MDM2, MDM4, and p53 levels. Both MMRi62 and MMRi67 induced p53 protein accumulation in NALM6 cells. However, only MMRi62 induced downregulation of MDM2 and MDM4 protein expression (**Figure 3A**). As expected, the MMRi62-induced downregulation of MDM2 and MDM4 occurred at posttranscriptional levels since the *MDM2* and *MDM4* mRNA levels were not altered by the treatment (**Figures 3B** and **S2A**). Similar results were also obtained in MV4-11 cells (**Figure S2B**). We then asked whether the MMRi62-induced MDM4 downregulation is an MDM2-dependent process. Because of technical difficulties during the establishment of stable MDM2-knockdown NALM6 cell lines, we decided to use a pair of MDM2-high MANCA lymphoma cells in which MDM2 was stably knocked down by lentivirus-mediated microRNA (miRNA) expression in comparison with a stable line established with control miRNA. Our results indicated that MDM4 expression levels were elevated in MDM2-knockdown MANCA-mlp-MDM2 cells, and treatment with DMSO, MMRi67, and MMRi62 did not induce MDM4 degradation. The elevated MDM4 expression in MDM2-knockdown cells is consistent with the report that MDM2 promotes ubiquitination and degradation of MDM4 (36). Abolishment of MDM4 degradation by MMRi62 in the absence of MDM2 in MANCA-mlp-MDM2 cells led us to conclude that MMRi62-induced MDM4 degradation requires MDM2. Notably, MMRi62 induced MDM2 degradation in NALM6 cells but not in MANCA cells at 5 μM (**Figures 3A, C**), thus suggesting that other cell-specific factors expressed in NALM6 but not MANCA cells are required for MMRi62-induced MDM2 degradation. Next, we attempted rescue experiments using proteasome inhibitor bortezomib in NALM6 cells but failed to reach a conclusion since these cells are quite sensitive to proteasome inhibitors such as bortezomib with IC50s in the nanomolar range (data not shown). Hence, we experimented with melanoma A375 cells, and our results showed that treatment with MMRi62 for 24 h induced MDM4 downregulation in a concentration-dependent manner; furthermore, we observed that this activity could be rescued by using a 1 μM bortezomib treatment for 16 h before cell harvest (**Figure 3D**). These results led us to conclude that the MMRi62-induced MDM4 degradation was proteasome dependent. To obtain evidence that MMRi62 increases ubiquitination of MDM2B and MDM4 in cells, we performed *in vivo* ubiquitination assays (21) using 293T cells co-transfected with MDM2B and MDM4 with His-ubiquitin followed by 24-h treatment with MMRi62 or MMRi67. The ubiquitinated proteins were pulled down by His-tag affinity purification with Nickel beads and then analyzed by WB analysis. As expected, MMRi62 increased ubiquitinated species of MDM2B and MDM4 (**Figure 3E**, smearing ladders of protein bands above MDM2B or MDM4 bands), which is consistent with the result that only MMRi62 induced proteasomal degradation of MDM4 and MDM2 proteins in leukemia cells (**Figure 3A**).

### MMRi62 induced apoptosis in a p53-independent manner

**Figure 4:**
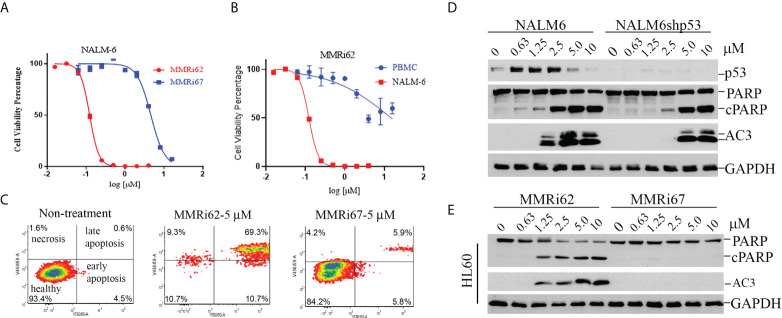
MMRi62 but not MMRi67 inhibited the proliferation of leukemic cells by inducing p53-independent apoptosis with low toxicity for healthy peripheral blood monocytes (PBMCs). **(A)** Growth curves of wt-p53 NALM6 cells in the presence of different concentrations of MMRi62 or MMRi67 in 72-h proliferation assays. **(B)** Growth curve of PBMCs in the presence of different concentrations of MMRi62 in comparison with that of NALM6 cells replotted from the same dataset in panel **(A)** PBMC proliferation was stimulated by 10 μg/ml of pokeweed mitogen (PWM). **(C)** Flow cytometry analysis of annexin-V-positive apoptotic cells after 48-h treatment with 5 mM of MMRi62 or MMRi67. **(D)** Concentration-dependent caspase-3 activation (AC3) and PARP cleavage (cPARP) in NALM6 cells and NALM6shp53 cells stably expressing shRNA against p53. The cells were treated with the indicated concentrations of either MMRi62 or MMRi67 for 24 h followed by Western blotting (WB) analysis with corresponding antibodies. **(E)** Similar analysis as in panel **(D)** except using p53-null HL60 cells.

Our results from anti-proliferation assays using p53-wt NALM6 and MV4-11 cells showed that MMRi62 was much more potent than MMRi67 in inhibiting leukemic cell proliferation. NALM6 cells had a much higher sensitivity to MMRi62 (IC50, ~0.12 µM) than MMRi67 (IC50, ~4.58 µM), as indicated by the 38-fold difference in their IC50s (**Figure 4A**) and a 7.8-fold difference in IC50s for MV4-11 cells (data not shown). We then tested how MMRi62 inhibits the growth of normal white blood cells using peripheral blood mononuclear cells (PBMCs). These assays were performed with proliferating PBMCs stimulated by 10 µg/ml of pokeweed mitogen. We obtained an IC50 of ~15 µM for MMRi62 in the PBMCs (**Figure 4B**), which indicates that leukemic NALM6 cells were ~125-fold more sensitive than PBMCs to MMRi62 (**Figure 4B**). Then, we investigated the aptitude and mechanisms for MMRi62- and MMRi67-induced apoptosis in leukemic cells. Flow cytometry analysis revealed that late-stage annexin-V-positive apoptotic cells increased from 0.6% to 69% with MMRi62 but only to ~6% with MMRi67 after 24-h treatment with the compounds at a concentration of 5 µM (**Figure 4C**). Consistent with the sharp difference in their anti-proliferation activity, MMRi62 potently induced caspase-3 activation, and PARP cleavage occurred at as low as ~1 µM after 24-h treatment and as early as 4 h after 1-µM treatment (**Figure S3A**). We then asked whether MMRi62-induced apoptosis is p53 dependent and performed WB assays using NALM6 cells and NALM6shp53 cells in which p53 was stably knocked down. Our results showed that MMRi62 induced a high level of caspase-3 activation and PARP cleavage in NALM6shp53 cells, although at a slightly diminished level as compared with NALM6 cells (**Figure 4D**), thus indicating that the MMRi62-induced apoptosis was largely p53 independent. Similar experiments showed that the MMRi67-induced apoptosis was p53 dependent (**Figure S3B**). To corroborate this conclusion, we repeated the same treatment in two p53-null leukemic cell lines, namely, HL60 and Jurkat cells. As expected, only MMRi62 induced significant caspase-3 activation and PARP cleavage in both cell lines (**Figures 4E** and **S3C**). These data suggest that MMRi62 inhibits leukemic cell growth *via* p53-independent apoptosis.

### MMRi62 induced apoptosis in drug-resistant leukemia cells and primary patient leukemia samples

**Figure 5:**
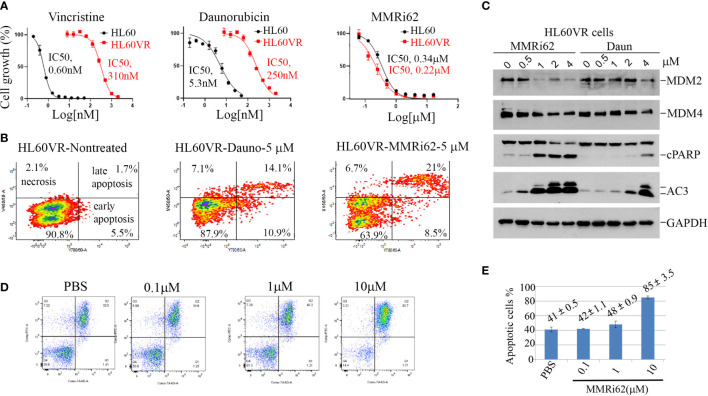
Results demonstrating that MMRi62 is a potent apoptosis inducer in acquired drug-resistant leukemia cells. **(A)** Growth curves of p53-null HL60 and HL60VR cells in the presence of different concentrations of vincristine (left), daunorubicin (middle), and MMRi62 (right). IC50s of each drug for the two cell lines are shown beside the corresponding curves. **(B)** Flow cytometry analysis of annexin-V-positive apoptotic cells in non-treated cells (left) or cells treated with 5 uM of daunorubicin (middle) or 5µM of MMRi62 (right) for 48 h. **(C)** Western blotting analysis of MDM2 and MDM4 expression, caspase-3 activation, and apoptotic PARP cleavage in HL60VR cells treated for 24 h at the indicated concentrations of MMRi62 or daunorubicin (Daun). **(D)** Flow cytometry analysis of annexin-V-positive apoptotic cells after 72-h treatment using an acute myeloid leukemia (AML) patient sample (Patient#02-1919) with the indicated concentrations of MMRi62. **(E)** Quantitative graph of the apoptotic fractions in different treatments in panel **(D)**.

In addition to intrinsic resistance to chemotherapy-induced apoptosis conferred by a p53 mutation, acquired resistance to chemotherapy also contributes to poor outcomes and recurrences after first-line treatment of leukemia. A vincristine-resistant leukemic HL60 cell line (HL60VR) was generated by continuous exposure to vincristine, and cells developed a multidrug-resistant phenotype because of the increased expression of MDR1 gene (37, 38). Our results showed that HL60VR was 516-fold resistant to vincristine compared with HL60 (**Figure 5A**, left) and was also 47-fold resistant to daunorubicin (**Figure 5A**, middle). Encouragingly, HL60VR and HL60 showed similar sensitivities to MMRi62 with IC50s of 0.34 µM for HL60 and 0.22 µM for HL60VR (**Figure 5A**, right). Flow cytometry analysis indicated that MMRi62 induced ~30% apoptosis (sum of early and late apoptotic cells), while daunorubicin induced ~25% apoptosis after 48-h treatment at 5 µM (**Figure 5B**). Analysis results for activated caspase-3 and PARP cleavage showed that MMRi62 was a better apoptosis inducer than daunorubicin in HL60VR cells treated at equal concentrations (**Figure 5C**). We then freshly established daunorubicin-resistant HL60 cells by pre-exposing them to a non-lethal low dose of daunorubicin (1× IC50 or IC75 dose) for 7 days, and cells were subsequently challenged by a 5× IC50 dose of daunorubicin or a 2.5× IC50 dose of MMRi62 followed by apoptosis analysis. Our results showed that pre-exposure to the low-dose daunorubicin was effective in establishing resistance in HL60 cells; during the challenge, these cells were killed by the 5× IC50 dose of daunorubicin but remained sensitive to apoptosis induction by the 2.5× IC50 dose of MMRi62 (**Figure S4**). These data suggest that MMRi62 bypasses the resistance mechanism acquired by pre-exposure to vincristine or daunorubicin and thus remains equally cytotoxic to these drug-resistant cells. Primary AML cells underwent spontaneous apoptosis when cultured *in vitro* for 72 h despite the fact that the medium was supplemented with multiple growth factors; however, treatment with 10 µM of MMRi62 increased the apoptotic fraction to 85%, a twofold enhancement from the phosphate-buffered saline (PBS)-treated samples (**Figure 5D**, compare PBS with 10 µM of MMRi62). These data suggest that MMRi62 targets/pathways do not overlap with those of vincristine or daunorubicin in leukemic cells, and the compound kills primary leukemic cells by apoptosis.

### MMRi62 induced apoptosis in primary patient leukemia samples and inhibited their colony formation *in vitro*

**Figure 6:**
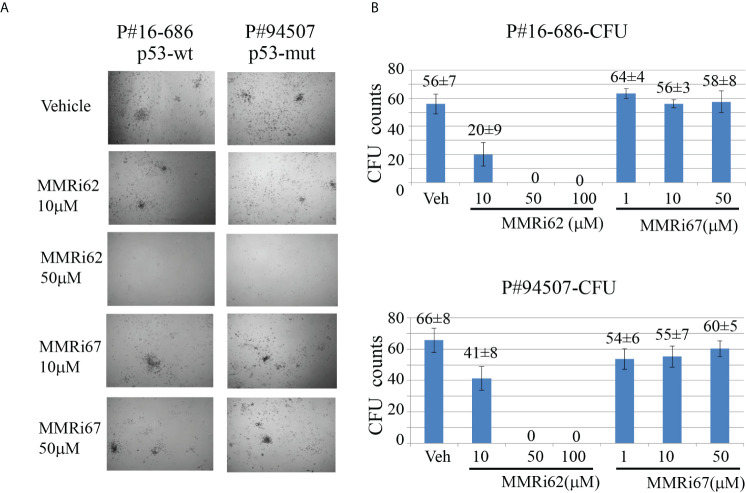
MMRi62 induced p53-independent apoptosis and inhibited colony-forming unit (CFU) formation in primary acute myeloid leukemia (AML) patient samples *in vitro*. **(A)** Representative images of CFU plates taken after 13 days of culture with primary AML patient samples pre-treated with the indicated concentrations of MMRi62 or MMRi67 for 4 h in a 37°C–5% CO_2_ incubator followed by culturing them in fully supplemented CFU growth media. **(B)** Quantitative graph of CFU numbers for two AML patient samples treated with either MMRi62 or MMRi67 at different concentrations.

To assess whether MMRi62 inhibits the survival of the leukemic progenitors with colony-forming unit (CFU) assays, primary AML samples were treated with different concentrations of MMRi62 or MMRi67 for 4 h and then plated in a semisolid methylcellulose medium containing several stem cell growth factors key to the growth of different progenitor cells for CFU to form in 12–15 days. MMRi62 but not MMRi67 inhibited CFU in a dose-dependent manner (**Figures 6A, B**). Importantly, the ability of MMRi62 to inhibit CFU was p53 independent, since IC50s for CFU inhibition were very similar with a value of ~12 µM for p53-wt and ~13 µM for p53-mutant AML patient samples (**Figure S5**). Taken together, these results suggest that MMRi62 is an active agent that can kill primary AML cells and their proliferating progenitors *in vitro*, regardless of their p53 status.

## Discussion

The development of MDM2–p53 disruptors for cancer therapy has been a major focus in the field for decades, and currently, several such inhibitors including MI-77301 (SAR405838), MK-8242 compound (SCH-900242), and AMG232 are under clinical trials (15). However, MDM4 overexpression (39, 40) confers intrinsic resistance to these inhibitors. We were the first to attempt to target the MDM2-MDM4 E3 complex as an alternative strategy to alter the oncogenic MDM2-MDM4 complex and previously reported that MMRi64 induces p53 accumulation for preferential apoptosis induction in p53-wt leukemia/lymphoma cells (30). In a secondary cell-based screen and characterization of MMRi6 analogs, this study has identified MMRi62 as an MDM4 degrader with potent pro-apoptotic capability. Interestingly, despite small structural differences, the mechanisms of action for MMRi62 and MMRi67 were quite different; i.e., MMRi62 inhibited MDM2-MDM4 E3 ligase not through dissociating the RING–RING interaction (**Figure 2A**) or inhibiting the polyubiquitination of the heterodimers (**Figure 1C**, polyUb) but by switching the preference of ubiquitination to MDM4 from MDM2 to promote MDM4 degradation and apoptosis in cells at <10 µM, whereas MMRi67 appeared to inhibit heterodimer formation resulting in inhibition of the E3 ligase activity of MDM2-MDM4 but with poor pro-apoptotic activities (**Figures 1**, **3A, C**, and **S3**). These results establish that there is a positive association between MDM4-degrader properties and pro-apoptotic activities in MMRi6 analogs. Although MDM4 degradation was found to be tightly associated with apoptosis induction in leukemic cells, the cause–result relationship could not be established because of technical difficulties in creating MDM4 knockdown cell lines. It is possible that there are other pro-apoptotic mechanisms involved in MMRi62-induced apoptosis. MMRi62 induces MDM4 degradation in leukemia/lymphoma cells in an MDM2-dependent manner but p53-independent manner (**Figures 3C** and **S3C**), which is consistent with MMRi6’s ability to preferentially promote MDM4 ubiquitination *in vitro* (**Figures 1** and **3**). Therefore, MMRi62 is a functional MDM4 inhibitor or an MDM4 degrader. We previously proposed a biochemical model in which ubiquitination of each component of the MDM2/MDM4/p53 ternary complex is a dynamic process where mutual competition for the acceptance of ubiquitin transfer and preference for ubiquitination/degradation changes depending on the relative ratio of the three components (24). Consistent with this model, MMRi62 treatment (within 5 µM) generated a unique protein degradation pattern: MMRi62 downregulated MDM2/MDM4 in a concentration-dependent manner but induced p53 accumulation inversely at higher concentrations of MMRi62 (**Figure 3A**). We interpret these results as MMRi62 having a dual mode of action depending on the concentration, i.e., lower concentrations of MMRi62 preferentially ubiquitinated MDM4/MDM2 resulting in the inhibition of p53 polyubiquitination and leading to p53 protein accumulation, while high concentrations of MMRi62 may have induced degradation of all three proteins (**Figure 3A**, **5** and 10 μM) *via* a different mechanism that requires further investigation. The results from thermal stability experiments (**Figure S6A**) showed that both MMRi62 and MMRi67 have similar dissociating potentials on preformed RING heterodimers at 5 and 10 µM since they induced a similar 3°C thermoshift from 68°C for the dimethyl sulfoxide (DMSO) control to 65°C. However, only MMRi67 inhibited *de novo* RING heterodimer formation in solution (**Figure 2A**). Further, MMRi62 is apparently more of a MDM4 degrader in cells than a MDM2 degrader because its MDM2 degradation activity varied in NALM6 and MANCA cells (**Figures 3A, C**).

The finding that MMRi62-induced apoptosis is p53 independent gives MMRi62 an advantage over MDM2/MDM4-p53 disruptors because MDM2/MDM4-p53 inhibitors strictly depend on p53 function for their antitumor activity. In addition to the challenges of acquired resistance caused by p53 mutation for MDM2–p53 disruptors (41, 42), non-mutational wtp53 dysfunction that occurs broadly in AML poses another challenge to these p53-based inhibitors (43). Thus, MMRi62-type compounds provide an opportunity for treating leukemia with either p53 deficiency or dysfunctional wtp53. Eliminating MDM4 and MDM2 by MMRi62 hits the key drug targets in AML because MDM4 is a pervasive driver of leukemogenesis in multiple mouse leukemia models (44), and MDM2 overexpression induced by MTF2 loss is a mediator for refractory AML (45). However, whether MMRi62 selectively kills MDM2-high or MDM4-high leukemia cells needs to be verified in the future. Whether MDM4 is the major determinant of MMRi62 sensitivity in leukemia cells could not be determined because of technical difficulties in obtaining shMDM4 leukemia cells. However, we found that MDM4 expression is a determinant of MMRi62 sensitivity in melanoma cells in which efficient shMDM4 knockdown was achieved (Lama et al., unpublished data), thus suggesting that MDM4 is one of the key cellular targets for MMRi62-induced apoptosis. Although MMRi62 induces lysosomal degradation of ferritin heavy chain 1 (FTH1) and triggers ferroptosis in pancreatic cancer cells (46), FTH1 degradation is not involved in MMRi62-induced apoptosis in leukemia cells since MMRi67 induced the same level of FTH1 degradation as MMRi62 but without incurring apoptosis (Lama et al., unpublished data). The mechanism underlying MMRi62-induced p53-independent apoptosis is currently unknown. We speculate that MDM4 elimination by MMRi62 unleashes all substrates of the oncogenic MDM2-MDM4 heterodimers in addition to p53 to confer pro-apoptotic activities. We previously reported that these MDM2 isoforms form hyperactive heterodimer E3 ligases with MDM4 that regulate p53 and MDM4 stability in cells (28). MDM2 splice isoforms MDM2A and MDM2B that lack p53-binding domains promote lymphomagenesis as efficiently as full-length MDM2 (47, 48). Therefore, MMRi62-induced MDM4 degradation will inactivate the E3 ligase activity of MDM2-MDM4, and potentially the E3 ligase activities of MDM2A-MDM4 and MDM2B-MDM4 heterodimers delivering the antitumor activity. Although our preliminary tests in HL60VCRluc-transplant mouse models showed that MMRi62 inhibited the HL60VCRluc burden *in vivo* but failed to prolong survival at the current formulation/route/schedule (**Figure S6B**), MMRi62 optimization may lead to the eventual development of a better MDM4 degrader that will be useful for refractory p53-deficient leukemia patients.

## Data availability statement

The original contributions presented in the study are included in the article/**Supplementary material**. Further inquiries can be directed to the corresponding author.

## Ethics statement

All animal experiments have been conducted in accordance with an IACUC protocol (Protocol number: 1276M) approved by Institutional Animal Care and Use Committee (IACUC) of Roswell Park Comprehensive Cancer Center.

## Author contributions

RL, CX, JQ-G, DM, SP, CM, FR, JW, and MG performed the biological experiments. SG and SC synthesized MMRi62 and MMRi67 and performed the chemical characterizations of these compounds. All authors contributed intellectually to this study. XW and RL wrote and revised the manuscript, and all authors approved the information in the manuscript. All authors contributed to the article and approved the submitted version.

## Funding

This work was supported in part by the National Institutes of Health grants R01CA208352 (XW, IGM) and GM078383 (SRC), the Roswell Park Alliance Foundation (RPAF) (XW, EW), and the RPAF Developmental Therapeutic Program Seed Funds (XW, SRC). We thank Alexander Fish for helping with the microscale thermophoresis (MST) experiments performed at NKI’s Protein Facility (The Netherlands) and supported by iNEXT-Discovery grants H2020-iNEXT PID:1662 and H2020-iNEXT-Discovery PID:12429 (IGM). This work was also supported by the National Cancer Institute (NCI) grant P30CA016056 involving the use of Roswell Park Comprehensive Cancer Center’s Shared Resources.

## Acknowledgments

We thank the Small Molecule Screening Shared Resource for the initial high-throughput screening of MMRi preliminary hits, the Translational Imaging Shared Resource (TISR) for BLI imaging, the Hematologic Procurement Shared Resource for providing human AML samples, and the Scientific Editing and Research Communications Core (SERCC) for editorial support. We thank Yuping Wang for their technical assistance. We also thank Dr. Deanna E. Conners for editing the manuscript.

## Conflict of interest

The authors declare that the research was conducted in the absence of any commercial or financial relationships that could be construed as a potential conflict of interest.

## Publisher’s note

All claims expressed in this article are solely those of the authors and do not necessarily represent those of their affiliated organizations, or those of the publisher, the editors and the reviewers. Any product that may be evaluated in this article, or claim that may be made by its manufacturer, is not guaranteed or endorsed by the publisher.

## References

[1] Freireich EJ Wiernik PH Steensma DP. The leukemias: A half-century of discovery. J Clin Oncol (2014) 32(31):3463–9. doi: 10.1200/JCO.2014.57.1034 25185093

[2] Kantarjian HM Keating MJ Freireich EJ. Toward the potential cure of leukemias in the next decade. Cancer (2018) 124(22):4301–13. doi: 10.1002/cncr.31669 30291792

[3] Desikan SP Daver N DiNardo C Kadia T Konopleva M Ravandi F. Resistance to targeted therapies: Delving into FLT3 and IDH. Blood Cancer J (2022) 12(6):91. doi: 10.1038/s41408-022-00687-5 35680852 PMC9184476

[4] Arai Y Chi S Minami Y Yanada M. FLT3-targeted treatment for acute myeloid leukemia. Int J Hematol (2022). doi: 10.1007/s12185-022-03374-0 35532877

[5] Montesinos P Recher C Vives S Zarzycka E Wang J Bertani G. Ivosidenib and azacitidine in idh1-mutated acute myeloid leukemia. N Engl J Med (2022) 386(16):1519–31. doi: 10.1056/NEJMoa2117344 35443108

[6] Siegel RL Miller KD Fuchs HE Jemal A. Cancer statistics, 2022. CA Cancer J Clin (2022) 72(1):7–33. doi: 10.3322/caac.21708 35020204

[7] Hannun YA. Apoptosis and the dilemma of cancer chemotherapy. Blood (1997) 89(6):1845–53. doi: 10.1182/blood.V89.6.1845 9058703

[8] Hanahan D Weinberg RA. Hallmarks of cancer: the next generation. Cell (2011) 144(5):646–74. doi: 10.1016/j.cell.2011.02.013 21376230

[9] Miyashita T Reed JC. Bcl-2 oncoprotein blocks chemotherapy-induced apoptosis in a human leukemia cell line. Blood (1993) 81(1):151–7. doi: 10.1182/blood.V81.1.151.151 8417786

[10] Frankfurt OS Byrnes JJ Seckinger D Sugarbaker EV. Apoptosis (programmed cell death) and the evaluation of chemosensitivity in chronic lymphocytic leukemia and lymphoma. Oncol Res (1993) 5(1):37–42. 8369574

[11] Carter BZ Milella M Tsao T McQueen T Schober WD Hu W. Regulation and targeting of antiapoptotic XIAP in acute myeloid leukemia. Leuk: Off J Leuk Soc America Leuk Res Fund UK (2003) 17(11):2081–9. doi: 10.1038/sj.leu.2403113 12970762

[12] Glaser SP Lee EF Trounson E Bouillet P Wei A Fairlie WD. Anti-apoptotic mcl-1 is essential for the development and sustained growth of acute myeloid leukemia. Genes Dev (2012) 26(2):120–5. doi: 10.1101/gad.182980.111 PMC3273836 22279045

[13] Cassier PA Castets M Belhabri A Vey N. Targeting apoptosis in acute myeloid leukaemia. Br J Cancer (2017) 117(8):1089–98. doi: 10.1038/bjc.2017.281 PMC5674101 29017180

[14] Aubrey BJ Kelly GL Janic A Herold MJ Strasser A. How does p53 induce apoptosis and how does this relate to p53-mediated tumour suppression? Cell Death Differ (2018) 25(1):104–13. doi: 10.1038/cdd.2017.169 PMC5729529 29149101

[15] Tisato V Voltan R Gonelli A Secchiero P Zauli G. MDM2/X inhibitors under clinical evaluation: perspectives for the management of hematological malignancies and pediatric cancer. J Hematol Oncol (2017) 10(1):133. doi: 10.1186/s13045-017-0500-5 28673313 PMC5496368

[16] Zenz T Benner A Dohner H Stilgenbauer S. Chronic lymphocytic leukemia and treatment resistance in cancer: The role of the p53 pathway. Cell Cycle (2008) 7(24):3810–4. doi: 10.4161/cc.7.24.7245 19098429

[17] Metzeler KH Herold T Rothenberg-Thurley M Amler S Sauerland MC Gorlich D. Spectrum and prognostic relevance of driver gene mutations in acute myeloid leukemia. Blood (2016) 128(5):686–98. doi: 10.1182/blood-2016-01-693879 27288520

[18] Takahashi K Patel K Bueso-Ramos C Zhang J Gumbs C Jabbour E. Clinical implications of TP53 mutations in myelodysplastic syndromes treated with hypomethylating agents. Oncotarget (2016) 7(12):14172–87. doi: 10.18632/oncotarget.7290 PMC4924706 26871476

[19] Richter-Pechanska P Kunz JB Hof J Zimmermann M Rausch T Bandapalli OR. Identification of a genetically defined ultra-high-risk group in relapsed pediatric T-lymphoblastic leukemia. Blood Cancer J (2017) 7(2):e523. doi: 10.1038/bcj.2017.3 28157215 PMC5386337

[20] Hav M Libbrecht L Ferdinande L Pattyn P Laurent S Peeters M. MDM2 gene amplification and protein expressions in colon carcinoma: Is targeting MDM2 a new therapeutic option? Virchows Arch (2011) 458(2):197–203. doi: 10.1007/s00428-010-1012-7 21113617

[21] Rekhi B Karnik N Agrawal R Shetty O Patkar S. Detection of MDM2 gene amplification on tissue microarray-based fluorescence in-situ hybridization (FISH) in well-differentiated and dedifferentiated liposarcomas, displaying a wide morphological spectrum: A validation study at a tertiary cancer referral centre. Indian J Pathol Microbiol (2022) 65(1):65–75. doi: 10.4103/IJPM.IJPM_1238_20 35074968

[22] Arnoff TE El-Deiry WS. MDM2/MDM4 amplification and CDKN2A deletion in metastatic melanoma and glioblastoma multiforme may have implications for targeted therapeutics and immunotherapy. Am J Cancer Res (2022) 12(5):2102–17. PMC9185629 35693093

[23] Konopleva M Martinelli G Daver N Papayannidis C Wei A Higgins B. MDM2 inhibition: an important step forward in cancer therapy. Leuk: Off J Leuk Soc America Leuk Res Fund UK (2020) 34(11):2858–74. doi: 10.1038/s41375-020-0949-z 32651541

[24] Wang X Wang J Jiang X. MdmX protein is essential for Mdm2 protein-mediated p53 polyubiquitination. J Biol Chem (2011) 286(27):23725–34. doi: 10.1074/jbc.M110.213868 PMC3129153 21572037

[25] Itahana K Mao H Jin A Itahana Y Clegg HV Lindstrom MS. Targeted inactivation of Mdm2 RING finger E3 ubiquitin ligase activity in the mouse reveals mechanistic insights into p53 regulation. Cancer Cell (2007) 12(4):355–66. doi: 10.1016/j.ccr.2007.09.007 17936560

[26] Huang L Yan Z Liao X Li Y Yang J Wang ZG. The p53 inhibitors MDM2/MDMX complex is required for control of p53 activity *in vivo* . Proc Natl Acad Sci U S A (2011) 108(29):12001–6. doi: 10.1073/pnas.1102309108 PMC3141917 21730163

[27] Pant V Lozano G. Limiting the power of p53 through the ubiquitin proteasome pathway. Genes Dev (2014) 28(16):1739–51. doi: 10.1101/gad.247452.114 PMC4197966 25128494

[28] Fan C Wang X. Mdm2 splice isoforms regulate the p53/Mdm2/Mdm4 regulatory circuit *via* RING domain-mediated ubiquitination of p53 and Mdm4. Cell Cycle (2017) 16(7):660–4. doi: 10.1080/15384101.2017.1288327 PMC5397270 28166445

[29] Meenalakshmi Chinnam CX Lama R Zhang X Cedeno CD Wang Y Stablewski AB. MDM2 E3 ligase activity is essential for p53 regulation and cell cycle integrity. PLoS Genet (2022) 18(5):e1010171. doi: 10.1371/journal.pgen.1010293 35588102 PMC9119546

[30] Wu W Xu C Ling X Fan C Buckley BP Chernov MV. Targeting RING domains of Mdm2-MdmX E3 complex activates apoptotic arm of the p53 pathway in leukemia/lymphoma cells. Cell Death Dis (2015) 6:e2035. doi: 10.1038/cddis.2015.358 26720344 PMC4720891

[31] Godar S Ince TA Bell GW Feldser D Donaher JL Bergh J. Growth-inhibitory and tumor- suppressive functions of p53 depend on its repression of CD44 expression. Cell (2008) 134(1):62–73. doi: 10.1016/j.cell.2008.06.006 18614011 PMC3222460

[32] Rosso M Polotskaia A Bargonetti J. Homozygous mdm2 SNP309 cancer cells with compromised transcriptional elongation at p53 target genes are sensitive to induction of p53-independent cell death. Oncotarget (2015) 6(33):34573–91. doi: 10.18632/oncotarget.5312 PMC4741474 26416444

[33] Kanizsai I Madacsi R Hackler L Jr. Gyuris M Szebeni GJ Huzian O. Synthesis and cytoprotective characterization of 8-hydroxyquinoline betti products. Molecules (2018) 23(8):1–25. doi: 10.3390/molecules23081934 PMC6222637 30072653

[34] Joseph P Errico IT Sills M Ong J Park F Allocco J. Assignee. METHODS AND COMPOSITIONS OF TARGETED DRUG DEVELOPMENT. (2013).

[35] Chou TC. Theoretical basis, experimental design, and computerized simulation of synergism and antagonism in drug combination studies. Pharmacol Rev (2006) 58(3):621–81. doi: 10.1124/pr.58.3.10 16968952

[36] Pan Y Chen J. MDM2 promotes ubiquitination and degradation of MDMX. Mol Cell Biol (2003) 23(15):5113–21. doi: 10.1128/MCB.23.15.5113-5121.2003 PMC165735 12860999

[37] Belhoussine R Morjani H Gillet R Palissot V Manfait M. Two distinct modes of oncoprotein expression during apoptosis resistance in vincristine and daunorubicin multidrug-resistant HL60 cells. Adv Exp Med Biol (1999) 457:365–81. doi: 10.1007/978-1-4615-4811-9_39 10500812

[38] Baran Y GU Ural AU. Expression of multidrug resistance (MDR1) gene in human promyelocytic leukemia cell line selected with vincristine. Turkish J Cancer (2005) 35(2):88–92.

[39] Patton JT Mayo LD Singhi AD Gudkov AV Stark GR Jackson MW. Levels of HdmX expression dictate the sensitivity of normal and transformed cells to nutlin-3. Cancer Res (2006) 66(6):3169–76. doi: 10.1158/0008-5472.CAN-05-3832 16540668

[40] Hu B Gilkes DM Farooqi B Sebti SM Chen J. MDMX overexpression prevents p53 activation by the MDM2 inhibitor nutlin. J Biol Chem (2006) 281(44):33030–5. doi: 10.1074/jbc.C600147200 16905541

[41] Hoffman-Luca CG Ziazadeh D McEachern D Zhao Y Sun W Debussche L. Elucidation of acquired resistance to bcl-2 and mdm2 inhibitors in acute leukemia *in vitro* and *in vivo* . Clin Cancer Res (2015) 21(11):2558–68. doi: 10.1158/1078-0432.CCR-14-2506 PMC4957562 25754349

[42] Jung J Lee JS Dickson MA Schwartz GK Le Cesne A Varga A. TP53 mutations emerge with HDM2 inhibitor SAR405838 treatment in de-differentiated liposarcoma. Nat Commun (2016) 7:12609. doi: 10.1038/ncomms12609 27576846 PMC5013668

[43] Prokocimer M Molchadsky A Rotter V. Dysfunctional diversity of p53 proteins in adult acute myeloid leukemia: Projections on diagnostic workup and therapy. Blood (2017) 130(6):699–712. doi: 10.1182/blood-2017-02-763086 28607134 PMC5659817

[44] Ueda K Kumari R Schwenger E Wheat JC Bohorquez O Narayanagari SR. MDMX acts as a pervasive preleukemic-to-acute myeloid leukemia transition mechanism. Cancer Cell (2021) 39(4):529–47.e7. doi: 10.1016/j.ccell.2021.02.006 33667384 PMC8575661

[45] Maganti HB Jrade H Cafariello C Manias Rothberg JL Porter CJ Yockell-Lelievre J. Targeting the mtf2-mdm2 axis sensitizes refractory acute myeloid leukemia to chemotherapy. Cancer Discov (2018) 8(11):1376–89. doi: 10.1158/2159-8290.CD-17-0841 PMC7200079 30115703

[46] Li J Lama R Galster SL Inigo JR Wu J Chandra D. Small-molecule mmri62 induces ferroptosis and inhibits metastasis in pancreatic cancer *via* degradation of ferritin heavy chain and mutant p53. Mol Cancer Ther (2022) 21(4):535–45. doi: 10.1158/1535-7163.MCT-21-0728 PMC10258866 35131878

[47] Steinman HA Burstein E Lengner C Gosselin J Pihan G Duckett CS. An alternative splice form of Mdm2 induces p53-independent cell growth and tumorigenesis. J Biol Chem (2004) 279(6):4877–86. doi: 10.1074/jbc.M305966200 14612455

[48] Fridman JS Hernando E Hemann MT de Stanchina E Cordon-Cardo C Lowe SW. Tumor promotion by Mdm2 splice variants unable to bind p53. Cancer Res (2003) 63(18):5703–6. 14522887

