# Supplementary figures and images for: Small molecule MMRi62 targets MDM4 for degradation and induces leukemic cell apoptosis regardless of p53 status
Source: Front Oncol. 2022 Aug 5;12:933446. doi: 10.3389/fonc.2022.933446 (PMC9389462; doi:10.3389/fonc.2022.933446)

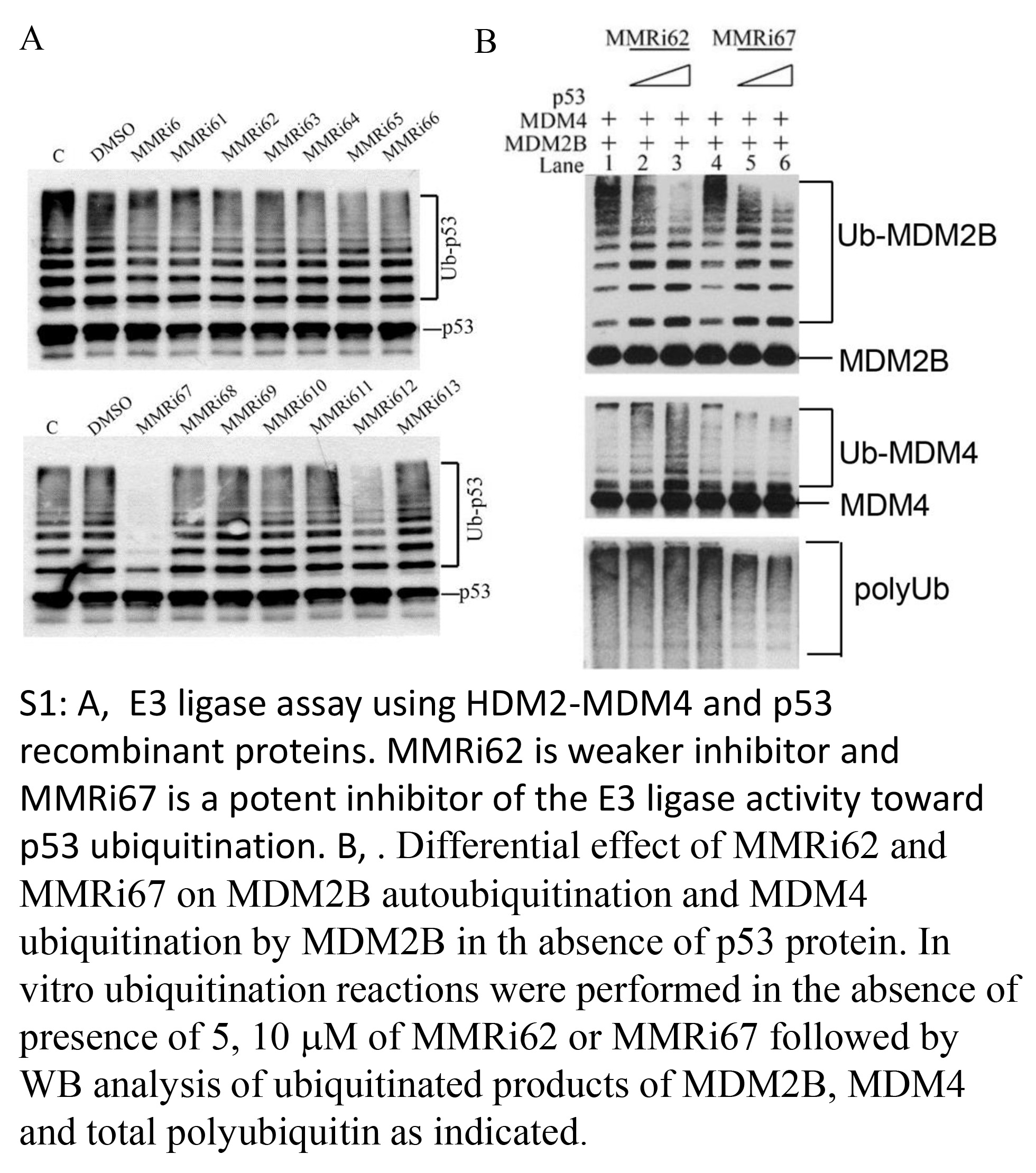

Supplement: Supplementary file 1 [file Image_1.jpeg]

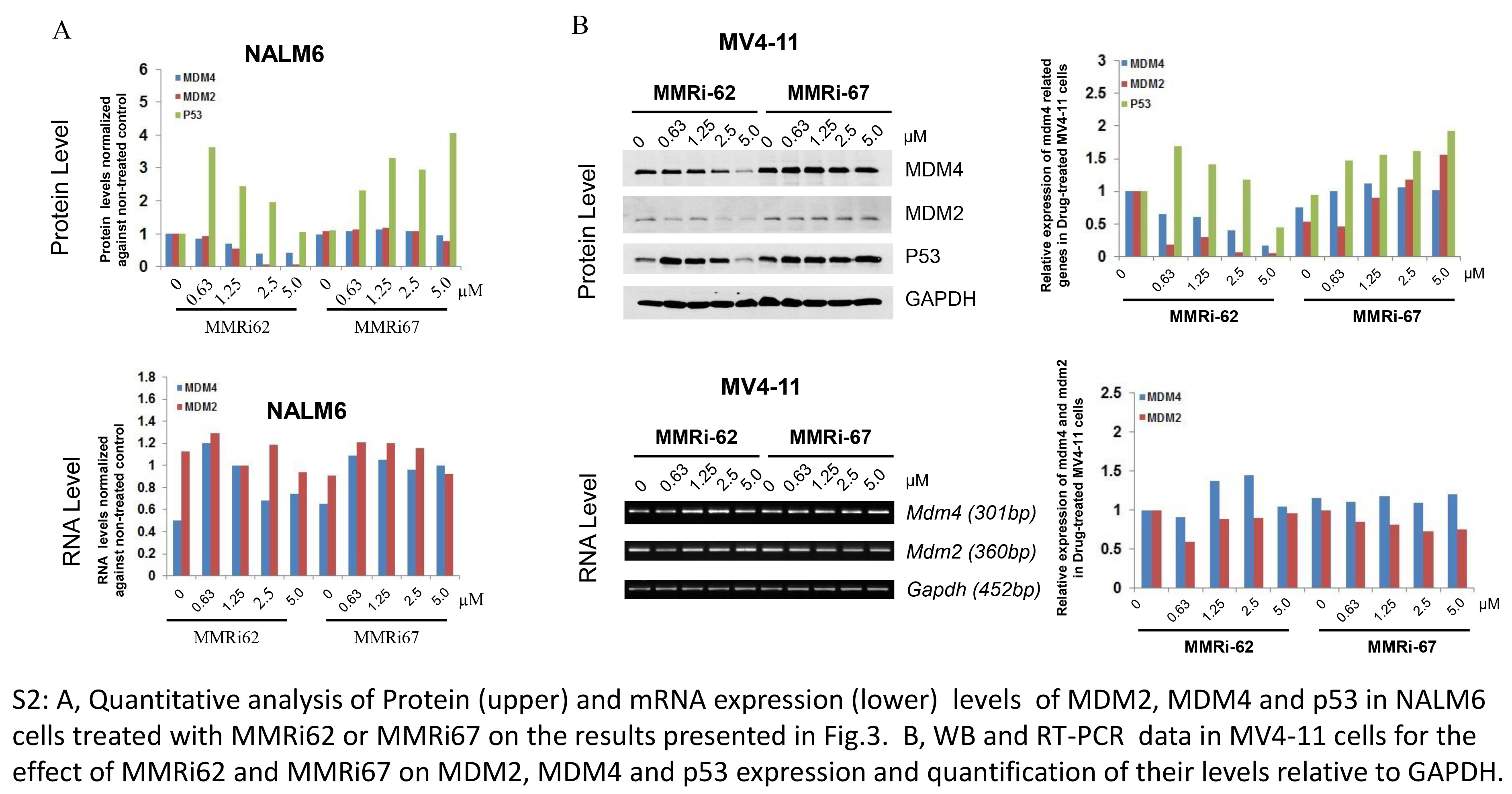

Supplement: Supplementary file 2 [file Image_2.jpeg]

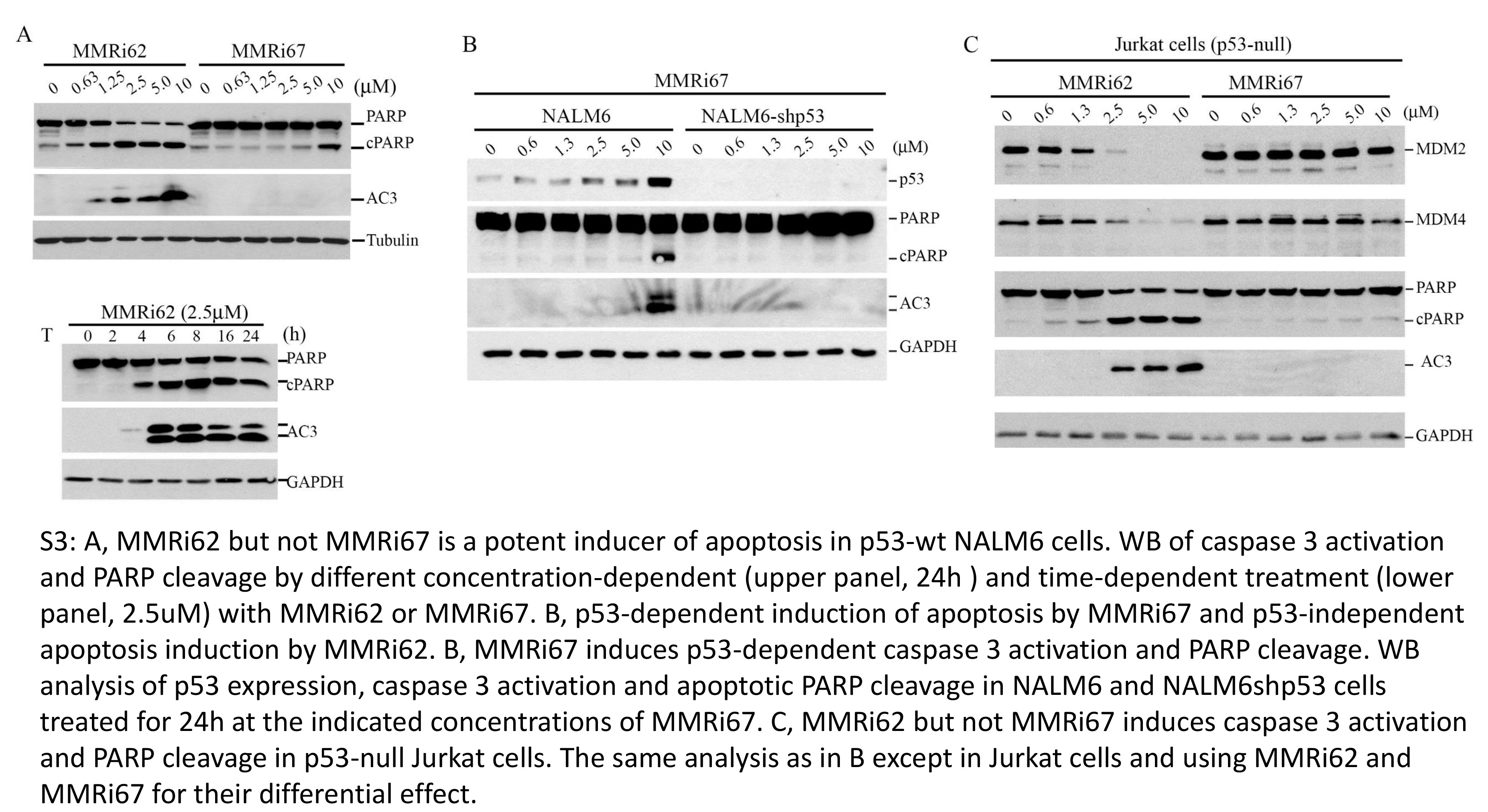

Supplement: Supplementary file 3 [file Image_3.jpeg]

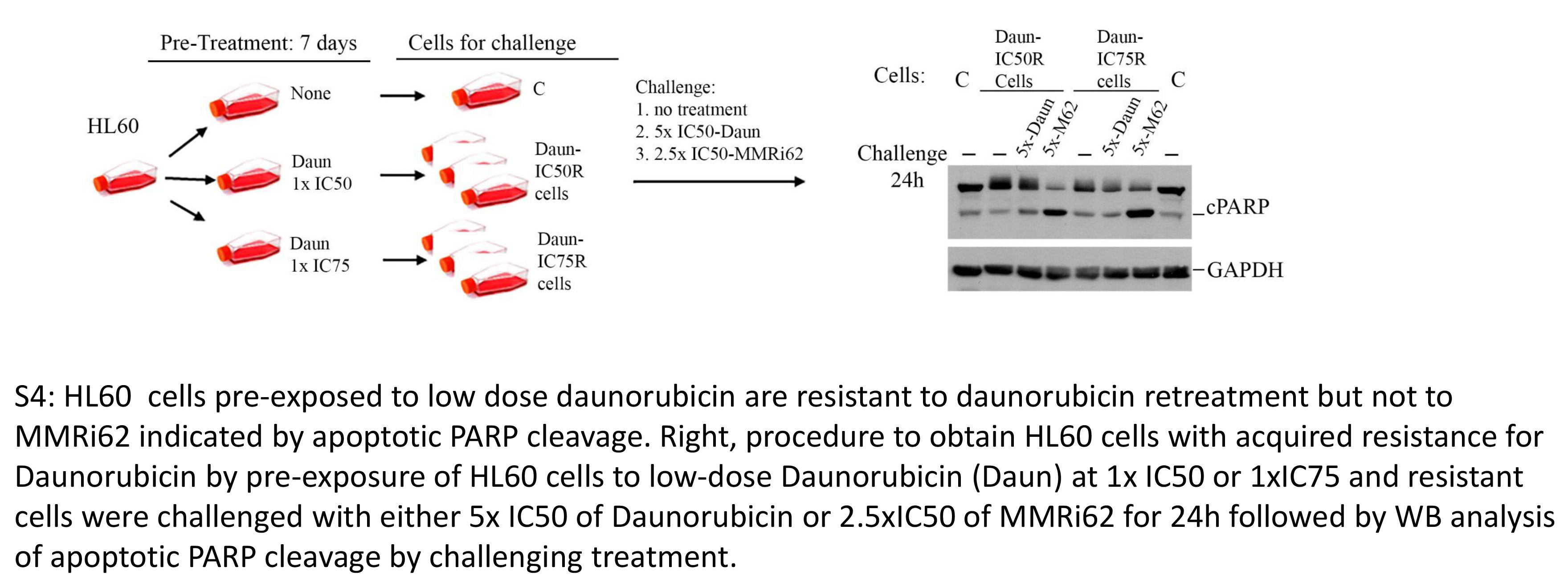

Supplement: Supplementary file 4 [file Image_4.jpeg]

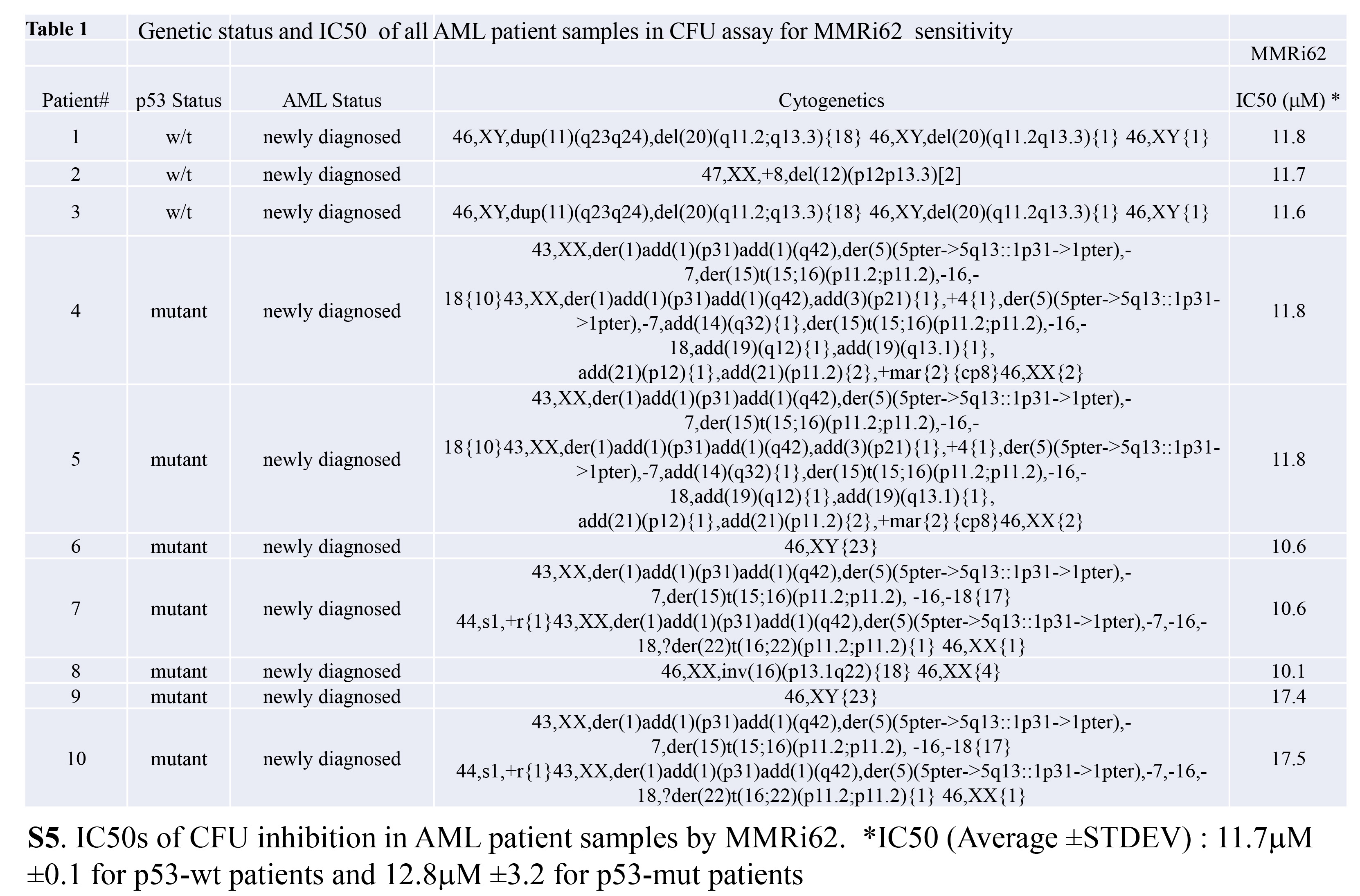

Supplement: Supplementary file 5 [file Image_5.jpeg]

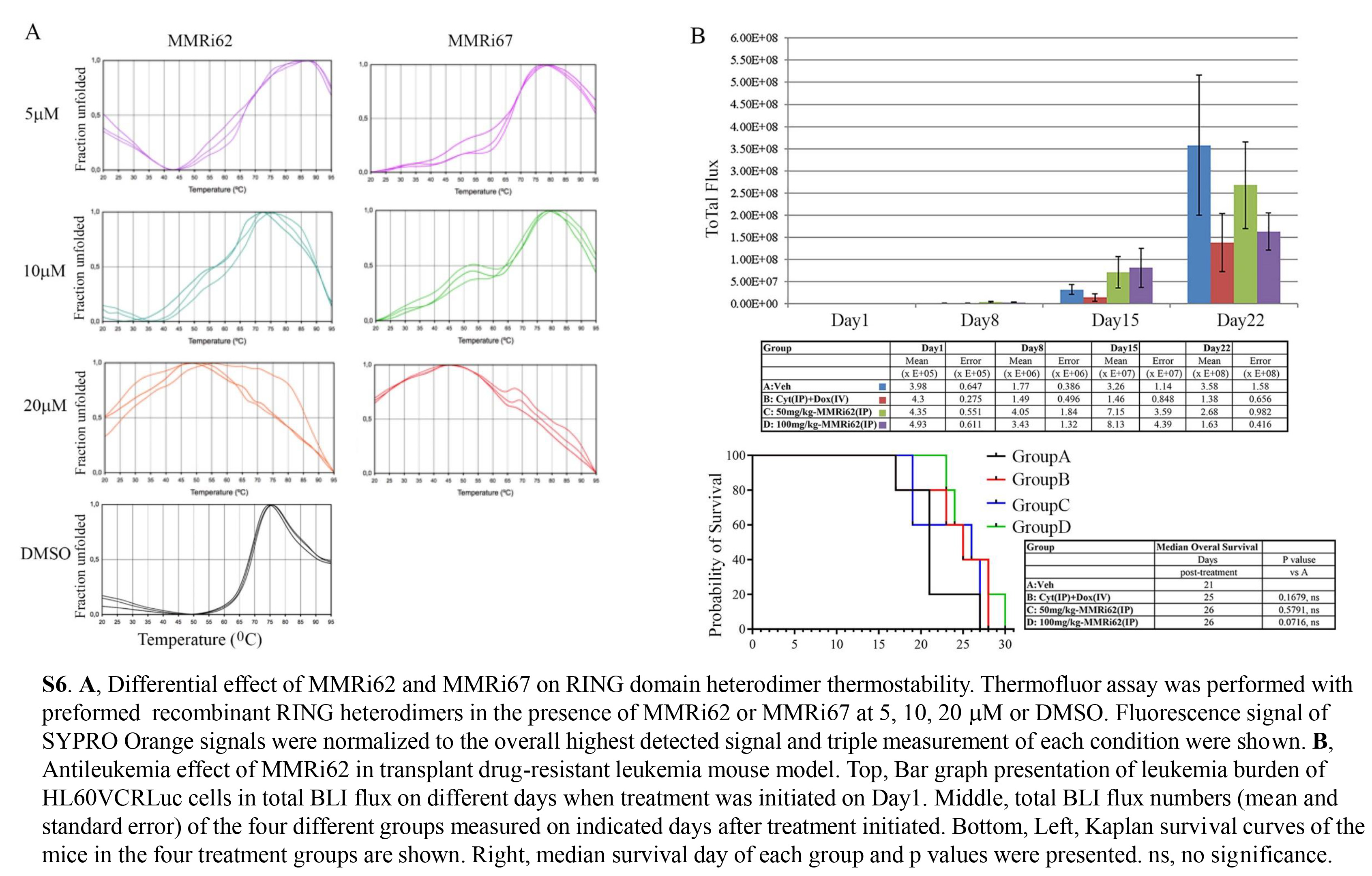

Supplement: Supplementary file 6 [file Image_6.jpeg]
