# Supplementary material for: Small molecule MMRi62 targets MDM4 for degradation and induces leukemic cell apoptosis regardless of p53 status
Source: Front Oncol. 2022 Aug 5;12:933446. doi: 10.3389/fonc.2022.933446 (PMC9389462; doi:10.3389/fonc.2022.933446)
Supplement: Supplementary file 7 [file Table_1.docx]

| **Supl Method**   \| **Antibody** \| **Company** \| **Catalogue #** \| \| --- \| --- \| --- \| \| p53 (DO-1) \| Santa Cruz Biotechnology \| sc-126 \| \| MDM2 (4B11) \| SIGMA-Millipore \| # MAB3776 \| \| MDM2(D1V2Z) \| Cell Signaling Technology \| # 86934S \| \| MDM4 \| Proteintech \| #17914-1-AP \| \| Anti-HA.11 Epitope Tag antibody \| BioLegend \| # 901514 \| \| PARP (FL and Cleaved) \| Cell Signaling Technology \| # 9532S \| \| GAPDH (FL-335) \| Santa Cruz Biotechnology \| sc-25778 \| \| Activated Caspase (ASP175) Antibody \| Cell Signaling Technology \| # 9661S \| | | | |  |  |
| --- | --- | --- | --- | --- | --- | --- | --- | --- | --- | --- | --- | --- | --- | --- | --- | --- | --- | --- | --- | --- | --- | --- | --- | --- | --- | --- | --- | --- | --- | --- | --- | --- |
|  | | | |  |  |
| **Primer** | **Oligo sequence** |  |  |  |  |
| MDM2-Forward | 5’-CACAAATCTGATAGTATTTC-3’ |  |  |  |  |
| MDM2-Reverse | 5’-CCAATAGTCAGCTAAGGA-3’ |  |  |  |  |
| MDM4-Forward | 5’-CTCCAAACTTTTTGATCCC-3 |  |  |  |  |
| MDM4-Reverse | 5’-CTGAATCTCTTTCTTGCAAAT-3’ |  |  |  |  |
| GAPDH-Forward | 5’-ACCACAGTCCATGCCATCAC-3’ |  |  |  |  |
| GAPDH-Reverse | 5’-TCCACCACCCTGTTGCTGTA-3’ |  |  |  |  |
